# Supplementary material for: Complexome profiling of the Chlamydomonas psb28 mutant reveals TEF5 as an early PSII assembly factor
Source: Plant Cell. 2025 Mar 17;37(6):koaf055. doi: 10.1093/plcell/koaf055 (PMC12164590; doi:10.1093/plcell/koaf055)
Supplement: koaf055_Supplementary_Data [file koaf055_supplementary_data.zip › psb28_interactive.zip!/plots/intensity/qualityPlots/peptides/RPP0_Cre12.g520500.t1.1.html]

### Cre12.g520500.t1.1

Trivial name: RPP0  
SeqCoverage [median]: 17.50  
Mass: 34.58 kDa  
  
 

| Sequence | MissedCleavage | Mass [Da] | wtIntensity | muIntensity | Score |
| --- | --- | --- | --- | --- | --- |
| ALRPGAVILMGK | 1 | 1224.74 | 0.00 | 557.06 | 45.43 |
| LNQYLQTYDK | 0 | 1284.63 | 0.00 | 326.56 | 84.21 |
| VGAVAPEDVVIK | 0 | 1195.68 | 658.54 | 2629.75 | 82.75 |
| VGPSQATLLAK | 0 | 1083.63 | 646.56 | 0.00 | 73.50 |
| VIESGAVYDPK | 0 | 1176.60 | 0.00 | 384.39 | 62.82 |
|  |  |  | 1305.1 | 3897.8 |  |
